# Peer PrEP referral + HIV self-test delivery for PrEP initiation among young Kenyan women: study protocol for a hybrid cluster-randomized controlled trial

**DOI:** 10.1186/s13063-023-07734-x

**Published:** 2023-11-04

**Authors:** Njeri Wairimu, Rachel C. Malen, Adriana M. Reedy, Peter Mogere, Irene Njeru, Carlos Culquichicón, Maureen McGowan, Fei Gao, Jared M. Baeten, Kenneth Ngure, Katrina F. Ortblad

**Affiliations:** 1 Partners in Health and Research Development, Center for Clinical Research, Kenya Medical Research Institute, Nairobi, Kenya; 2 Public Health Sciences Division, Fred Hutchinson Cancer Center, Seattle, USA; 3 Department of Epidemiology, University of Washington, Seattle, USA; 4 Heidelberg Institute of Global Health, Heidelberg University, Heidelberg, Germany; 5 Vaccine and Infectious Diseases Division, Fred Hutchinson Cancer Center, Seattle, USA; 6 Department of Global Health, University of Washington, Seattle, USA; 7 Department of Medicine, University of Washington, Seattle, USA; 8 School of Public Health, Jomo Kenyatta University of Agriculture and Technology, Nairobi, Kenya

**Keywords:** HIV prevention, Pre-exposure prophylaxis (PrEP), Adolescent girls and young women (AGYW), Peer referral, Self-testing, Kenya

## Abstract

**Background:**

Oral HIV pre-exposure prophylaxis (PrEP) for HIV prevention is highly effective, but uptake remains low in Africa, especially among young women who are a priority population for HIV prevention services. HIV self-testing (HIVST) has been proven to increase HIV testing in diverse populations but has been underutilized to support linkage to HIV prevention services. Most young women who initiate PrEP in Africa do so through informal peer referral. We wanted to test a model of formalized peer referral enhanced with HIVST delivery among young Kenyan women.

**Methods:**

The Peer PrEP Trial is a two-arm hybrid effectiveness-implementation cluster-randomized controlled trial being conducted in central Kenya. Eligible participants (i.e., peer providers, *n* = 80) are women (≥ 16–24 years) refilling or initiating PrEP at public healthcare clinics who can identify at least four peers who could benefit from PrEP and not enrolled in another HIV study. Peer providers will be 1:1 randomized to (1) *formal peer PrEP referral* + *HIVST delivery*, where they will be encouraged to refer four peers (i.e., peer clients, ≥ 16–24 years) using educational materials and HIVST kits (two per peer client), or (2) *informal peer PrEP referral*, where they are encouraged to refer four peer clients using informal word-of-mouth referral. In both arms, peer providers will deliver a standard PrEP referral card with information on nearby public clinics delivering PrEP services. Peer providers will complete surveys at baseline and 3 months; peer clients will complete surveys at 3 months. Our primary outcome is PrEP initiation among peer clients, as reported by peer providers at 3 months. Secondary outcomes include PrEP continuation (any refilling), HIV testing (past 3 months), sexual behaviors (past month), and PrEP adherence (past month) among peer clients, as reported by both peer providers and clients at 3 months. Implementation outcomes will include participants’ perceived acceptability, appropriateness, and feasibility of the intervention as well assessments of the intervention’s fidelity and cost.

**Discussion:**

Evidence from this trial will help us understand how HIVST could support health systems by facilitating linkage to PrEP services among young women who could benefit in Kenya and similar settings.

**Trial registration:**

ClinicalTrials.gov NCT04982250. Registered on July 29, 2021.

**Supplementary Information:**

The online version contains supplementary material available at 10.1186/s13063-023-07734-x.

## Introduction

Pre-exposure prophylaxis (PrEP) for HIV prevention is highly effective [1–5] but uptake and continuation remain low among young African women, a priority population for the delivery of HIV prevention services [6]. In Kenya, a country with the fourth largest HIV epidemic worldwide [7, 8], the government began delivering oral PrEP on a national scale in 2017 [9, 10]. One of the priority groups for HIV incidence reduction for the Kenya Ministry of Health is adolescent girls and young women (AGYW, ≥ 16–24 years), who account for 33% of the total new HIV infections in Kenya yet comprise only 10% of the population [11–13]. Barriers to PrEP initiation for this population are multi-faceted and include community (e.g., stigma associated with use) and intra-personal (e.g., lack of PrEP knowledge or self-efficacy) factors [6, 14, 15]. Thus, innovative models for PrEP delivery that can help overcome these barriers are needed.

The World Health Organization has recommended HIV self-testing (HIVST) as a strategy to increase HIV testing [16], but the tool has been underutilized to support health systems. Many studies have demonstrated that HIVST increases recent and frequent HIV testing among diverse populations (e.g., female sex workers [FSWs], HIV serodiscordant couples, men, and women) and settings, including Kenya [17–21]. However, few interventions have explored how HIVST could facilitate linkage to HIV services, and those that have primarily focused on linkage to treatment services [22–25]. Most people who test for HIV test, however, will test negative; thus, HIVST, when packaged with other appropriate implementation strategies, has great potential to support linkage to HIV prevention services, including PrEP.

The opinion of peers often influences the behaviors and preferences of young women, including behaviors related to the uptake of health services, such as contraception [26–29]. Peer referrals and peer-delivered interventions have been demonstrated to be feasible and effective among populations with tight social connectivity (e.g., men who have sex with men, FSWs) to increase identification of new individuals living with HIV and facilitate linkage to treatment interventions [30–32]. In Kenya, most AGYW initiate PrEP following informal (i.e., word-of-mouth) referral from peers [33]. A model of formalized peer PrEP referral enhanced with HIVST delivery could help increase PrEP initiation among this population at an increased risk of HIV acquisition.

Formative qualitative research conducted by this team found that young Kenyan women would be willing to engage in a peer PrEP referral model supported with HIVST delivery [33]. Specifically, young women engaged in PrEP services anticipated they would be willing to educate their peers about PrEP use and safety, deliver and assist peers with HIVST, and support peers with linkage to PrEP services and PrEP adherence, if needed. With input from Kenyan stakeholders (including young female PrEP users and non-users) during a one-day stakeholder meeting, we designed such a model for formal testing [34]. A hybrid effectiveness-implementation randomized-controlled trial aims to measure the effect of this intervention on PrEP initiation among AGYW while assessing implementation outcomes to expedite the translation of research into practice [35].

## Methods

This paper has been developed in accordance to the Standard Protocol Items: Recommendations for Interventional Trials (i.e., SPIRIT) reporting guidelines [36] (Table 1).

**Table 1:** SPIRIT reporting guidelines for interventional trials

	**Study period**			
	**Enrollment**	**Baseline**	**Post-allocation**	
**Timepoint**		**0 months** ***Providers***	**3 months** ***Providers***	**3 months** ***Clients***
**Enrollment:**				
Eligibility screen^a^	X			X
Informed consent	X			X
Peer provider randomization	X			
Peer providers refer peer clients^b^		X		
**Interventions:**				
*Formal peer PrEP referral* + *HIVST delivery*		X		
*Informal peer PrEP referral*		X		
**Assessments:**				
*Baseline characteristics*^*c*^		X		X
*Primary and secondary outcomes*^*d*^			X	X

*Abbreviations*: *HIVST* HIV self-testing, *PrEP* pre-exposure prophylaxis

^a^See Table 2 for more details on trial inclusion and exclusion criteria

^b^Peer providers will refer up to 4 peer clients within 3 months of baseline, after completion of the group training for those randomized to the intervention arm

^c^See Table 3 for details on baseline characteristic variables

^d^See Table 4 for details on primary and secondary outcome variables

### Study design and setting

The “Peer PrEP” trial is a 2-arm hybrid effectiveness-implementation cluster-randomized controlled trial (ClinicalTrials.gov: NCT04982250). The trial will occur in Central Kenya (Kiambu, Nairobi, and Murang’a counties), which has largely peri-urban and rural populations and a population-level HIV prevalence of ~ 4% [37]. The Partners in Health and Research Development (PHRD) team, located in Kiambu County, will conduct the trial. This team — which includes a site Principal Investigator (PI), study coordinator, nurse counselor, research assistants, recruitment officers, data coordinators, and laboratory technicians — has extensive experience conducting PrEP clinical and implementation research [1, 38–40], a strong history of community engagement with diverse populations, and experience working collaboratively with healthcare providers in the region [38, 41, 42].

### Study participants

The trial will enroll 2 types of participants: (1) *peer providers*, who are AGYW who have initiated PrEP and will refer peers to PrEP services, and (2) *peer clients*, who are AGYW who have not initiated PrEP and will be referred to PrEP services (Table 2). Eligible participants will be female, ≥ 16 to 24 years old (including emancipated minors ≥ 16 to 17 years — i.e., AGYW who are married, mothers, pregnant, or household heads [43–45]), and able and willing to provide consent and complete research activities. Eligible peer providers will be those who have initiated PrEP, can identify up to 4 peers who could benefit from PrEP, and are willing to be randomized to the intervention. Eligible peer clients will be those referred to PrEP by a peer provider. Peer providers will be ineligible if they are currently enrolled in another HIV-related study or are illiterate.

**Table 2:** Eligible criteria and recruitment strategies for trial peer provider and clients

Participant type	Eligibility	Ineligibility	Recruitment
**Peer providers** (*n* = 80)	• ≥ 16 to 24 years old^a^ • Female • Must have refilled or initiated PrEP (i.e., been dispensed PrEP) • Can identify four peers at HIV risk who could benefit from PrEP • Not currently enrolled in an HIV study • Able and willing to be randomized, participate in research activities, and provide informed consent	• Not ≥ 16 to 24 years old^a^ • Male • Have not used PrEP • Cannot identify four peers at HIV risk who could benefit from PrEP • Unable or willing to be randomized, participate in research activities, and provide informed consent • Currently enrolled in an HIV study • Illiterate	• Recruit participants from HIV clinics where PrEP is available using strategies established by the research team (e.g., workshops for healthcare workers)
**Peer clients** (*n* = 320 max)	• ≥ 16 to 24 years old^a^ • Female • Referred by peer provider to PrEP services (formally or informally) • Able and willing to participate in research activities and provide informed consent	• Not ≥ 16 to 24 years old^a^ • Male • Not referred by peer provider to PrEP services (formally or informally) • Not able or willing to participate in research activities and provide informed consent	• Peer providers will recruit peer clients • At the point of recruitment, peer clients will call research staff to provide their contact information for follow-up. If peer clients call the study staff, both the peer client and provider will receive 50 KES via mobile money

Abbreviations: *PrEP*, pre-exposure prophylaxis; *KES*, Kenyan Shillings

^a^AGYW aged 16–17 years will only be eligible if they are emancipated minors, i.e., are mothers, pregnant, married, or household heads

Peer providers will be recruited on a rolling basis by PHRD staff from public healthcare clinics delivering PrEP services in the region. Peer clients will be recruited by peer providers, who will be instructed to only recruit peers who meet the eligibility criteria. At the point of recruitment, peer providers will encourage peer clients to call study staff to share their contact information and schedule a study visit 3 months following recruitment. Peer providers and clients will each be compensated 50 Kenyan Shillings (KES; ~ $0.50 US Dollars (USD)) each time a client calls the study line; only peer clients who complete this activity will be contacted for follow-up.

All enrolled participants will complete informed written or verbal (select peer clients only) consent by experienced PHRD research assistants and receive 300 KES (~ $3 USD) for their engagement in research activities (i.e., completion of study surveys). For participants who visit the PHRD clinic to complete surveys in person, additional reimbursement for transportation will be provided.

### Study procedures

**Fig. 1:**
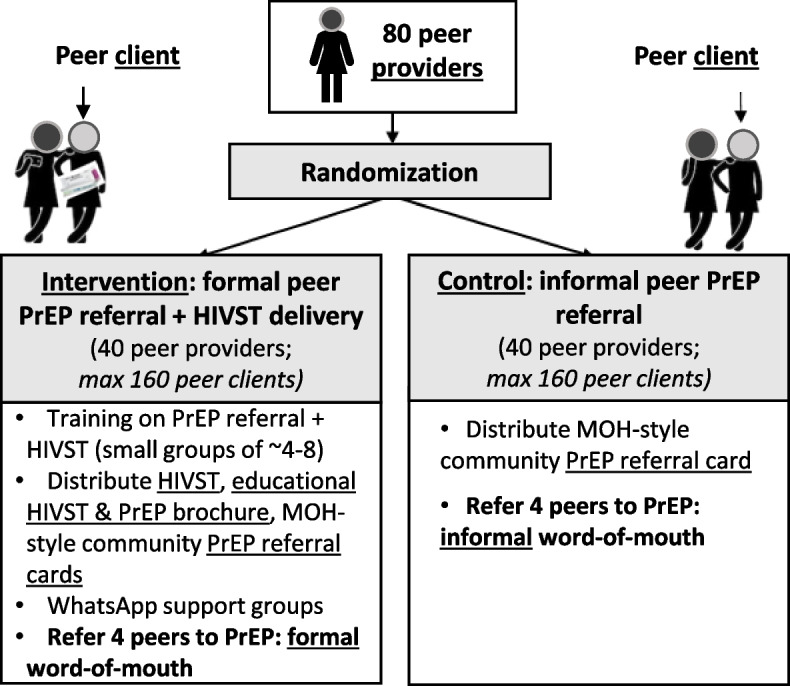
Design of the Peer PrEP two-arm cluster-randomized trial

Following enrollment, peer providers will be randomized 1:1 to either (1) *formal peer PrEP referral* + *HIVST delivery*, where peer providers will receive a brief training and be encouraged to refer four peers to PrEP using techniques from the training, PrEP and HIVST educational materials, and HIV self-tests, or (2) *informal peer PrEP referral*, where peer providers will be encouraged to refer 4 peers to PrEP using informal word-of-mouth referral techniques (Fig. 1).

#### Randomization

The randomization allocation sequence for this study will be generated using computer-generated random numbers and consistent block sizes without any stratification factors [46]. This sequence will be generated by the author C.C., who has no direct involvement with participants. Once a peer provider has enrolled in the study and completed informed consent, a PHRD research assistant will hand them an opaque, sealed envelope containing their study arm assignment. This envelope will be opened at the point of delivery so the research assistant can document the study arm assignment. Due to the inherent characteristics of the intervention, this trial is unblinded.

#### Intervention: Formal peer PrEP referral + HIVST delivery

Before peer providers identify and refer peer clients to PrEP services, they will complete a one-day, AGYW-friendly training. This training will occur in small groups (i.e., ~ 4–8 peer providers) on a rolling basis dependent on the pace of peer provider enrollment. This training will be led by PHRD research staff experienced in engaging AGYW on topics related to HIV prevention. The training will include information on PrEP use and safety, other available HIV prevention interventions, and guidance on HIVST use and results interpretation. Additionally, the training will include strategies for initiating conversations around PrEP and facilitating linkage to clinic-based HIV prevention or treatment services. At the training, peer providers will have the opportunity to use an HIVST kit with a fake buffer (i.e., water), which they can take and later use as a demonstration kit during peer referral. This training will emphasize the importance of maintaining peers’ confidentiality, not using stigmatizing language, and addressing potential HIV myths and stereotypes.

Following training, peer providers will receive a bag, designed to meet the style preferences of Kenyan AGYW, with the following intervention materials:

*Informational brochures (n* = *4).* The AGYW-friendly informational brochures will reinforce key facts about PrEP and HIVST use and safety. They will also outline steps that peer clients can follow to link to clinic-based confirmatory HIV testing and HIV prevention and treatment services, based on their HIVST result. A study phone number will also be included in these brochures that peer clients can call if they have any questions about PrEP or HIVST. Each bag will include 4 brochures, 1 for each referred peer client.

*HIVST kits (n* = *8).* This study will use the SURE CHECK® HIV 1/2 Assay (ChemBio Diagnostics, Medford, USA), a blood-based HIVST kit with a sensitivity of 99.7% and specificity of 99.9%, or a comparable blood-based HIVST kit approved by the World Health Organization [18]. These kits will come with written and pictorial instructions from the manufacturer (available in English) to help guide kit use and results interpretation. Each bag will include eight HIVST kits, two per each referred peer client. Peer providers will encourage peer clients to use 1 HIVST kit to test themselves and 1 kit to test with a sexual partner (if desired) or test again later.

*PrEP referral cards (n* = *4).* The PrEP referral cards will be modeled after standard Kenya Ministry of Health community PrEP referral cards and include information on the location of nearby public healthcare clinics with free HIV prevention and treatment services. Each bag will include 4 referral cards, 1 for each referred peer client.

The peer providers will be encouraged to refer four peer clients with known behaviors associated with HIV risk (e.g., condomless sex with partners of unknown HIV status, engagement in transactional sex) to PrEP services using the strategies gained and materials obtained from the peer provider training. The peer provider will be encouraged to assist peer clients with HIVST if the client is interested and both parties are comfortable. At the point of HIVST delivery, the peer provider will emphasize that the HIVST kits are for HIV screening and that confirmatory clinic-based HIV testing is necessary for PrEP initiation. Although not a requirement of the intervention, peer providers randomized to this arm will be encouraged to escort peers to clinics to facilitate linkage to HIV prevention or treatment services and support peers’ adherence to these services through informal check-ins, if they feel comfortable. Over the duration of the trial, all interested peer providers will have access to an optional WhatsApp group, monitored by PHRD research staff, where they can ask questions and get support with intervention delivery, if needed.

#### Control: Informal peer PrEP referral

Peer providers randomized to the control arm will also be encouraged to refer 4 peers with known behaviors associated with risk of HIV acquisition to clinic-based PrEP services. They will not receive any formalized training on PrEP and HIVST or peer referral, nor will they receive any HIVST kits for distribution to peers. The peer providers in this arm will be encouraged to talk to their peers about PrEP in the way they would typically have conversations about sexual behaviors and other health interventions (e.g., contraception). Like in the intervention arm, they will be given PrEP referral cards (the same as those described above) to distribute to their peers (4 cards in total, 1 per referred peer client).

If peer clients referred to PrEP services by peer providers in either the intervention or control arms initiate PrEP at nearby public healthcare clinics, they will receive standard-of-care PrEP services according to Kenya’s national PrEP implementation guidelines without any additional support from the study team [10].

### Study visits

**Fig. 2:**
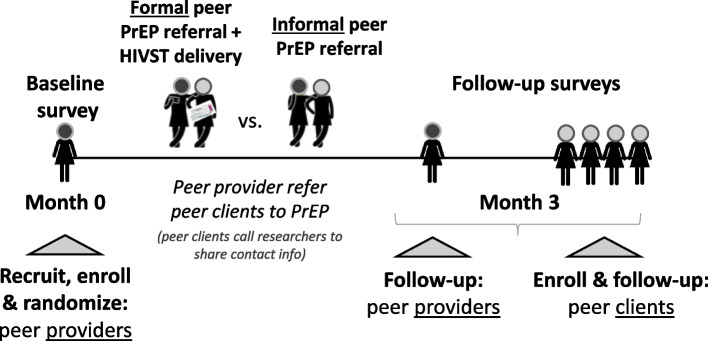
Overview of study visits and data collection activities

Over the duration of the trial, peer providers will complete 2 study visits and peer clients will complete 1 study visit (Fig. 2). All peer providers will complete a baseline (month 0) and follow-up (month 3) visit, while peer clients who contacted study staff at the point of recruitment will only complete a follow-up visit (month 3). This timeline should give interested peer clients referred to PrEP services sufficient time to both initiate PrEP and potentially refill PrEP 1 month following initiation.

### Data collection

Information on participants’ demographics (e.g., age, education) and behaviors associated with HIV risk will be collected in surveys conducted at baseline for peer providers and follow-up for peer clients (i.e., their first study visit) (Table 3). At the follow-up visit, self-reported outcomes related to delivery of the intervention and uptake of PrEP services among peer clients will be measured along with several implementation outcomes (described below). All surveys will be conducted by experienced PHRD researcher assistants using CommCare (Dimagi, Cambridge, USA), an electronic data collection tool.

**Table 3:** Data collection at the different study visits

Survey section	Description	Baseline	Follow-up	
		***Providers***	***Providers***	***Clients***
Demographics	Age, education, marital status, relationship status, age of sexual partner, monthly income	X		X
Sexual behaviors	Sexual intercourse frequency, condom usage, number of sexual partners, HIV status of partner, STI history, contraception usage, PEP usage, transactional sex, HIV testing history	X	X	X
Depression	Patient Health Questionnaire-2 (PHQ-2) scale	X	X	X
Relationship with peers	Sexual behavior discussion with peers, and peers' risk of HIV infection. Additionally, we will use the Multidimensional Scale of Perceived Social Support (MSPSS) scale	X	X	X
HIV risk perception, PrEP use, knowledge, and stigma	Perception of HIV risk in the next 3 months. PrEP usage history, PrEP initiation, adherence/retention, continuation, escorted peers to PrEP services, intervention materials delivered to peers. General PrEP knowledge. PrEP stigma based on the Modified Perceived Stigma Scale Kaai 2012	X	X	X
Self-efficacy and experience of Peer PrEP referral + HIVST model	Provider: set of questions asking provider's abilities to deliver the PrEP referral + HIVST model, and their experiences asking after delivering the model. Client: set of questions asking the client’s experiences on receiving the PrEP referral + HIVST model	X	X	X
Acceptability, appropriateness, and feasibility of Peer PrEP referral + HIVST model	Acceptability: Theoretical Framework of Acceptability (TFA). Appropriateness, and feasibility: Intervention Appropriateness Measure (IAM), and Feasibility of Intervention Measure (FIM) scales	X	X	X
Social harms	Gender-based violence and harms related to online PrEP referral + HIVST model	X	X	X

*Abbreviations*: *HIV*, human immunodeficiency virus; *HIVST*, HIV self-testing; *HPV*, human papillomavirus; *PrEP*, pre-exposure prophylaxis

Baseline surveys for peer providers will be conducted at the PHRD research clinic, the public clinics where peer providers are recruited, or other locations convenient to the peer providers. Follow-up surveys will be conducted either in-person at the PHRD research clinic or over the phone to potentially increase retention, especially among peer clients who that might have been recruited outside of Kiambu County. At follow-up, dried blood spot (DBS) samples will be collected to measure PrEP adherence from a random subset of 46 peer clients (23 per arm) who return to the PHRD research clinic for in-person follow-up visits.

### Outcomes

This hybrid effectiveness-implementation trial will measure effectiveness and implementation outcomes (Table 4).

**Table 4:** Trial outcome definitions and assessment timing

Outcomes	Definition	Denominator	Reported by	Timing
***Primary and secondary***				
PrEP initiation *(primary)*	% peer clients that went to a clinic and were dispensed PrEP	Peer clients referred	Peer providers^a^	Month 3
Recent HIV testing	% peer clients completed any HIV testing since referral	Peer clients referred	Peer providers^a^	Month 3
PrEP continuation	% peer clients returned to a clinic and refilled PrEP since referral	Peer clients on PrEP^b^	Peer providers^a^	Month 3
PrEP adherence	% peer clients report being adherent to PrEP using validated scales^c^; % with any TFV-DP detected in dried blood spot samples (random subset of participants)	Peer clients on PrEP^b^	Peer clients	Month 3
PrEP continuation (among peer providers)	% peer provider returned to a clinic to refill PrEP in the past 3 months	Peer providers	Peer providers	Month 3
***Implementation outcomes***				
Acceptability	% of peer clients and peer providers who report our intervention model is acceptable using validated scales^c^	Providers and referred clients	Providers and clients	Months 0 and 3
Appropriateness	% of peer clients and peer providers who report our intervention model is appropriate using validated scales^c^	Providers and referred clients	Providers and clients	Months 0 and 3
Feasibility	% of peer clients and peer providers who report our intervention model is feasible using validated scales^c^	Providers and referred clients	Providers and clients	Months 0 and 3
Fidelity	% of peer clients and peer providers who report delivering or receiving core components of the intervention (e.g., HIVST kits)	Providers and referred clients	Providers and clients	Month 3

*Abbreviations*: *HIVST*, HIV self-testing; *PrEP*, pre-exposure prophylaxis; *TFV-DP*, tenofovir-diphosphate

^a^Additionally reported by peer clients we were able to reach for follow-up; included as a sensitivity analysis

^b^Additionally reported by peer clients that initiated PrEP services (as reported by peer providers); included as a sensitivity analysis

^c^We used the following set of validated scales to measure their corresponding outcomes. Adherence: Self-Report Measure for Medication Adherence (Wilson 2016). Acceptability: Theoretical Framework of Acceptability (Sekhon 2017). Appropriateness: Intervention Appropriateness Measure scale (Weiner 2017). Feasibility: Feasibility of Intervention Measure scale (Weiner 2017)

#### Effectiveness outcomes

The primary trial outcome will be PrEP initiation among all referred peer clients, as reported by peer providers at month 3. PrEP initiation is defined as visiting a health clinic and being dispensed PrEP. Peer providers will report this outcome on behalf of peer clients due to anticipated challenges following up with referred peer clients (as experienced in a pilot study that tested this intervention [47]). In studies measuring secondary distribution models, it is common for outcomes among those receiving an intervention to be reported by those delivering the intervention [23, 48–50].

Secondary trial outcomes include any recent HIV testing among referred peer clients and any PrEP continuation and adherence among referred peer clients that initiated PrEP, all reported by peer providers except for the adherence outcomes. HIV testing will include any form of peer client HIV testing following referral to PrEP services by peer providers. PrEP continuation will be defined as a peer client returning to a clinic following PrEP initiation and being dispensed PrEP. PrEP adherence will include both self-reported and biological assessments; a 3-item validated scale will be used to assess self-reported adherence [51] and DBS samples will be used to assess biologic adherence (i.e., any detection of tenofovir diphosphate in a 3-mm punch [52–54]). Recent PrEP continuation (in the past 3 months) will also be assessed among peer providers.

#### Implementation outcomes

Peer providers’ and clients’ perceived acceptability, appropriateness, and feasibility of the peer PrEP referral + HIVST delivery model will be assessed, as will the fidelity of intervention delivery. The acceptability assessment will be based on the Theoretical Framework of Acceptability [55], which defines acceptability as a multidimensional construct comprised of seven components (e.g., burden, perceived effectiveness, affective attitude). The appropriateness and feasibility assessments will be based on the Intervention Appropriateness Measure [56] and Feasibility of Intervention Measure [56], respectively. For each outcome, clients will be asked to report how strongly they agree (using a 5-point Likert scale) with statements that assess different component constructs of these outcomes [55]; the percentage of participants that agree or strongly agree with the different statements will be reported. To measure fidelity, participants’ knowledge of PrEP and HIVST use and how many of the intervention core components they received or delivered (e.g., number of HIVST kits, number of PrEP referral cards — with variations by study arm) will be reported.

### Statistical analysis

Intention-to-treat, complete-case analyses will be used to compare differences in outcome proportions among peer clients between the randomization arms. Risk differences will be estimated with 95% confidence intervals for binary outcomes using mixed-effect models, with the study arm included as a fixed effect and the peer provider included as a random effect. A Gaussian distribution with an identity link will be used to fit the regression models and standard errors robust to clustering at the peer provider level will be used. Pre-specified sensitivity analyses include (1) estimating effect sizes for study outcomes as reported by peer clients (instead of peer providers), (2) study outcomes assessed among all potential referred peer clients (i.e., 4 per peer provider) and not just those referred, and (3) PrEP continuation and adherence outcomes among all peer clients referred to PrEP (not just those who initiated PrEP). Stata 16 (StataCorp, College Station, USA) will be used to run all analyses [57] and significance will be defined at the *p* = 0.05 level.

This trial was powered to detect significant differences in the primary outcome, PrEP initiation among peer clients at month 3. In a pilot study testing the peer PrEP referral + HIVST delivery model in Kiambu County, peer providers typically referred 75% of the suggested number of peer clients (*n* = 4) and PrEP initiation among peer clients referred was 81% (as reported by peer providers) [47]. If 63% of peer clients initiate PrEP with informal peer referral in this trial and there are 40 clusters (i.e., peer providers) per arm, three sampling units (i.e., peer clients) per cluster, and an intra-cluster correlation coefficient of 0.05 (typical for cluster-randomized controlled trials [46]), this trial has 80% power to detect a 17% difference in the proportion of peer clients initiating PrEP between the study arms. This would result in an overall sample of 80 peer providers and 320 peer clients (or 240 peer clients based on our assumption that peer providers will only refer 3 of the suggested 4 peer clients).

### Study oversight

#### Coordinating center

The Fred Hutchinson Cancer Center (Fred Hutch, Seattle, USA) serves as the coordinating center for the trial. The Fred Hutch team includes the study PI, project coordinators, a statistical research associate, and a biostatistics advisor. This team works collaboratively with the PHRD team (Thika, Kenya) to organize weekly team meetings (via Microsoft Teams), perform data quality checks for incoming trial data, monitor study implementation, develop solutions to any identified implementation challenges, ensure the approved institutional review board protocols and survey tools are up to date (requesting any modifications, as needed), and analyze study data (when the timing is appropriate). None of the Fred Hutch team members will have any direct involvement with study participants; all implementation activities (including participant recruitment and enrollment) are the responsibility of the PHRD team.

#### Data safety and monitoring board (DSMB)

This study will be overseen by an independent DSMB with members experienced in HIV prevention research in the region and implementation science. Every 6 months, the DSMB will review study conduct, progress, and related operational study data in open sessions, and study outcome data by arm in closed sessions. Additionally, the DSMB will review any reports of incident HIV infections, adverse events, and social harms by study arm. Adverse events will include any forms of verbal, physical, emotional, or economic abuse; serious adverse events will include hospitalization (for any condition, including those outside the study) or death. During study visits, participants will be asked to report if they have experienced any of these events and whether they were related to study participation. The DSMB will provide recommendations to the study team following each review, but no formal interim statistical evaluation will be conducted.

### Confidentiality

Every effort will be made to protect participants’ privacy and confidentiality. All survey data will be de-identified using a unique study identification number for each enrolled participant, enabling us to link data from individual participants over time. Any records (e.g., locator forms, consent forms) that contain personal identifiers of participants will be stored in a locked file in a room with limited access at the PHRD research site. All local databases will be secured with password-protected access systems.

### Dissemination

The study team is committed to public dissemination of the trial findings to participants, local Kenyan policymakers and stakeholders (e.g., members of the Kenya Ministry of Health, young women in the community at HIV risk, community advisory groups, and healthcare providers), and the global scientific community. Findings from this trial will be presented at arranged meetings, at local and international conferences, and in peer-reviewed academic journals.

## Discussion

PrEP has great potential to help curb the HIV epidemic; however, for it to achieve this objective, it needs to reach the populations that could benefit most and are not currently engaged in PrEP services [58]. AGYW are a priority population for the delivery of HIV prevention services in Africa, but their uptake of PrEP has been low [6, 59]. The peer PrEP referral + HIVST delivery model combines formalized peer referral and HIVST in a combination HIV prevention package to help increase PrEP uptake among this important population. It is based on evidence that peer-delivered health interventions can increase intervention uptake among populations with close social connectivity [30–32, 60–63], such as AGYW, and that HIVST can increase HIV testing uptake [18, 64, 65], which is along the care pathway to PrEP initiation and could help motivate this behavior among the vast majority of AGYW who will test HIV-negative.

This trial has strengths and limitations. Strengths include the use of HIVST to support health systems by motivating engagement in HIV prevention services. An additional strength includes formalizing the most common way AGYW are already coming into PrEP services — via informal word-of-mouth referral — to ensure they have accurate information on PrEP use, safety, and locations where they can initiate PrEP services. Potential trial limitations include relying on outcomes among peer clients reported by peer providers, but as discussed this is necessary because of anticipated challenges following up with peer clients. Additional limitations include limiting the number of peer clients peer providers are encouraged to recruit and only delivering two HIVST kits to each client. Findings from this trial could inform future adaptations to this model that could change some of these model details.

If this trial demonstrates the formalized peer PrEP referral + HIVST delivery intervention significantly increases PrEP initiation over informal PrEP referral among Kenyan AGYW, this could inform national and international PrEP implementation guidelines [10, 58, 66] on strategies that can be utilized to reach this important population. Additionally, this model could be adapted to increase initiation of other health services that could benefit this population, including newer longer-acting PrEP forms that will soon be available in Kenya (e.g., bi-monthly injectable cabotegravir) [67], human papillomavirus vaccination [68], and family planning services [69].

## Trial status

The first trial participant was enrolled on May 3, 2023 and it is anticipated the last trial participant will be enrolled around March 2024 (which will conclude follow-up with all peer providers and clients). This trial was first registered on ClinicalTrials.gov on July 29, 2021. All relevant ethics committees have approved the trial and any relevant modifications. At the time of this publication, the current protocol was version 1.10 (October 2023).

### Supplementary Information

**Additional file 1.**

## Data Availability

The trial protocol, data, and statistical code will be available upon request to study investigators by qualified researchers.

## Abbreviations

AGYW: Adolescent girls and young women
DSMB: Data Safety Monitoring Board
DBS: Dried blood spot
FSW: Female sex worker
HIV: Human immunodeficiency virus
HIVST: HIV self-testing
KES: Kenyan Shillings
PHRD: Partners in Health Research Development
PI: Principal Investigator
PrEP: Pre-exposure prophylaxis
SPIRIT: Standard Protocol Items: Recommendations for Interventional Trials
USD: United States Dollars
